# Genetic and Epigenetic Signatures in Acute Promyelocytic Leukemia Treatment and Molecular Remission

**DOI:** 10.3389/fgene.2022.821676

**Published:** 2022-04-12

**Authors:** Veronika Borutinskaitė, Andrius Žučenka, Aida Vitkevičienė, Mindaugas Stoškus, Algirdas Kaupinis, Mindaugas Valius, Eglė Gineikienė, Rūta Navakauskienė

**Affiliations:** ^1^ Department of Molecular Cell Biology, Life Sciences Center, Institute of Biochemistry, Vilnius University, Vilnius, Lithuania; ^2^ Hematology, Oncology, and Transfusion Medicine Centre, Vilnius University Hospital Santaros Klinikos, Vilnius, Lithuania; ^3^ Proteomic Center, Life Sciences Center, Institute of Biochemistry, Vilnius University, Vilnius, Lithuania

**Keywords:** blast, relapse, molecular remission, epigenetics, acute promyelocytic leukemia

## Abstract

Acute myeloid leukemia (AML) is an aggressive, heterogeneous group of malignancies with different clinical behaviors and different responses to therapy. For many types of cancer, finding cancer early makes it easier to treat. Identifying prognostic molecular markers and understanding their biology are the first steps toward developing novel diagnostic tools or therapies for patients with AML. In this study, we defined proteins and genes that can be used in the prognosis of different acute leukemia cases and found possible uses in diagnostics and therapy. We analyzed newly diagnosed acute leukemia cases positive for t (15; 17) (q22; q21) PML-RAR alpha, acute promyelocytic leukemia (APL). The samples of bone marrow cells were collected from patients at the diagnosis stage, as follow-up samples during standard treatment with all-trans retinoic acid, idarubicin, and mitoxantrone, and at the molecular remission. We determined changes in the expression of genes involved in leukemia cell growth, apoptosis, and differentiation. We observed that WT1, CALR, CAV1, and MYC genes’ expression in all APL patients with no relapse history was downregulated after treatment and could be potential markers associated with the pathology, thereby revealing the potential value of this approach for a better characterization of the prediction of APL outcomes.

## Introduction

AML is a very heterogeneous disease with regard to clinical features and acquired genetic alterations, with remission rates inversely related to age: 5-year survival in adults under 65 years old is 33% and in older adults (over 65) it is only 4% (Meyers et al., 2013). Acute promyelocytic leukemia (APL), the M3 subtype of acute myeloid leukemia (AML), accounts for 10% of all AML cases (McCulloch et al., 2017). The t (15; 17) translocation that generates PML-RARα fusion mRNA is detected in as many as 90% of APL patients and has become the definitive marker for the disease (Dos Santos et al., 2013; Zhang et al., 2021). The standard treatment protocols include combinations of anthracyclines (typically idarubicin) and all-trans retinoic acid (ATRA) with or without cytarabine depending on the patient’s age and health state. ATRA induces differentiation of APL cells and reduces the risk of bleeding and coagulation problems. However, APL still remains one of the most difficult subtypes of acute myeloid leukemia to treat because of its complications (Ades et al., 2010; Efficace et al., 2014; Hambley et al., 2021). Arsenic trioxide was discovered to be an effective agent in relapse cases. It was shown that arsenic trioxide was the most effective agent in treating APL (Ades et al., 2010; Efficace et al., 2014; Yilmaz et al., 2021).

Currently, cytogenetic and molecular markers according to the WHO system are used in the clinic to diagnose AML patients. However, these guidelines are not suitable for distinguishing all subtypes and do not always predict clinical outcomes (Dos Santos et al., 2013; Hunsu et al., 2021). APL relapse cases are very rare, but early diagnosis and the possibility of prediction of APL relapse are important because patients can develop bone marrow failure and life-threatening coagulopathies.

In the current work, we have studied the pattern of differential protein and gene expression of blasts from APL patients with relapse history in comparison to matched patients who enter remission due to finding possible prognostic molecular markers. We showed changes in the expression of proteins involved in the drug resistance process during the remission and relapse stages of APL. We observed that calreticulin (CALR), caveolin1 (CAV1), MYC, and WT1 can be potential markers associated with the pathology, thereby revealing the potential value of this approach for a better characterization of the prediction of APL outcomes.

## Materials and Methods

### Patient Samples, Blasts Phenotype, and Purification

Bone marrow samples from Lithuanian patients during 2018–2019 were collected after informed consent from patients at diagnosis and during treatment. All patients gave their written informed consent for genetic analysis and for the use of the laboratory results for scientific studies. The study was approved by the Ethics Committee of Biomedical Research of Vilnius District (No. 158200-16-824-356) and adhered to the tenets of the Declaration of Helsinki. Marrow samples from four normal donors collected at the time of donation for bone marrow transplantation were similarly collected and handled. AML phenotyping, cytogenetic, and molecular genetic analysis were carried out in the context of routine diagnostics at the Hematology, Oncology and Transfusion Medicine Centre, Vilnius University Hospital Santaros Klinikos (Tables 1, 2). Cells were analyzed freshly or after thawing of samples frozen in liquid nitrogen. Mononuclear cells were isolated by the Ficoll gradient. The fusion transcript level (%) of t (15; 17) (q22; q21) PML-RAR alpha was monitored using the RT-qPCR method as described earlier (Gabert et al., 2003).

**TABLE 1:** Clinical data of APL patient with a history of relapse.

	Patient (No.8)														
Month	0	4	7.5	9.5	12	13.5	14	14.5	15	15.5	16.5	18	19	20	21
Treatment	Diagnosis	ATRA +IDA	ATRA +IDA + MTX	MT	MT	MT	MT	MT	MT	Relapse	Trisenox	Trisenox	—	—	—
Gender	Female														
Bone marrow blasts, n (%)	47	<5	<5	<5	<5	<5	<5	<5	<5	22	23	<5	<5	<5	<5
White blood cells (×10^9^/L), n (%)	2.54	N	N	N	N	N	N	N	N	1.42	N	N	N	N	N
Platelets (×10^9^/L), n (%)	6	N	N	N	N	N	N	N	N	16	N	N	N	N	N
Hemoglobin (g/dl), n (%)	8.9	N	N	N	N	N	N	N	N	10.6	N	N	N	N	N
Survival (month)	Yes														
Immunophenotyping	CD45^+^, CD33^+^, CD123^+^, CD38^+^, CD71^+^, CD117^+^, CD13^+^, CD64^+^, CD123^+^, CD4^−^, CD2^−^, CD11b-, CD34^−^, CD56^−^, HLA-DR-														
BRC3	3.3E-01 positive for t (15; 17) (q22; q21) PML-RAR alpha	1E-03	NEG	NEG	NEG	4E-04	1.4E-03	1.5E-01	4.8E-03	2.1E-01	2.3E-01	1.5E-03	<1E-04	<1E-04	NEG
	Negative for inv (16) (p13; q22) CBFB-MYH11; t (8; 21) (q22; q22) AML1-ETO; NPM1; FLT3 ITD; CEBPA														

ATRA, all-trans retinoic acid; IDA, idarubicinum; MTX, mitoxantrone; MT, maintenance treatment with 6-mercaptopurine, methotrexate, ATRA; N, normal, WBC, white blood cells (× 109/L), n (%); PTL, platelets (× 109/L), n (%); HB, hemoglobin (g/dl), n (%); NEU, neutrophils (× 109/L), n (%).

**TABLE 2:** Summarized results of clinical data of APL patients with full molecular remission (patients 1–7).

	Patient 1	Patient 2	Patient 3	Patient 4	Patient 5	Patient 6	Patient 7
Gender	Female	Male	Female	Female	Male	Female	male
Age (years)	24	45	49	29	48	58	41
Bone marrow blasts, n (%)	73	81	23	81	85	85	82
White blood cells (×10^9^/L), n (%)	3.99	1.14	1.53	0.84	2.12	1.5	6.15
Platelets (×10^9^/L), n (%)	20	45	48	73	40	30	16
Hemoglobin (g/dl), n (%)	12,0	10,8	9,8	8.9	8.0	8.6	7.9
Survival	Yes						
Immunophenotyping	CD45^+^ dim, CD33^+^ bright, CD123+, CD38^+^, CD117+, CD13^+^ bright, CD64^+^, CD2^+^ dim, CD4^+^ dim, CD15^+^ dim, CD34^+^ dim, cMPO^+^ bright, HLA-DR-	CD45^+^ dim, CD33^+^ bright, CD123^+^, CD38^+^ dim, CD117^+^, CD13^+^ bright, CD64^+^ dim, CD56^+^ dim, cMPO^+^ bright, CD34^−^, HLA-DR-	CD45^+^ dim, CD33^+^ bright, CD123^+^, CD38^+^ dim, CD117^+^, cMPO^+^ bright, CD13^+^, CD2^+^, CD34^+^, HLA-DR-	CD45^+^ dim, CD33^+^ bright, CD123^+^, CD38^+^, CD117+ dim, CD13^+^ bright, CD64^+^, CD15^+^ dim, cMPO^+^ bright, CD34^−^, HLA-DR-	CD45^+^ dim, CD38^+^, CD117+, CD13 + het, CD33^+^, CD64^+^, CD123^+^, cMPO^+^, CD11b^+^, CD11c^+^ dim, CD15 + het, CD34^−^, HLA-DR-	CD45^+^ dim, CD33^+^ bright, CD123^+^, CD38^+^, CD117+ dim, CD13^+^ het, CD64^+^, CD15^+^ dim, cMPO^+^ bright, CD34^−^, HLA-DR-	CD45^+^ dim, CD117+, CD38^+^, CD2^+^ dim, CD4^+^ partial, CD11c+ dim, CD13^+^, CD33^+^, CD64^+^, CD99^+^ dim, CD123^+^, cMPO^+^, CD34^−^, HLA-DR-, CD11b-, CD14^−^, CD15^−^, CD25^−^, CD36^−^, CD56^−^, IREM2-
Cytogenetic	Positive for t (15; 17) (q22; q21) PML-RARalpha						
	BRC3 7.19E-01	BRC1 4.36E-01	BRC1 2.68E-01	BRC3 6.949E-01	BRC2 4.99E-01	BRC3 4.58E-01	
	negative for inv (16) (p13; q22) CBFB-MYH11; t (8; 21) (q22; q22) AML1-ETO; NPM1; FLT3 ITD; CEBPA						

ATRA, all-trans retinoic acid; IDA, idarubicin; MTX, mitoxantrone; MT, maintenance treatment with 6-mercaptopurine, methotrexate, ATRA; N, normal, WBC, white blood cells (× 10^9^/L), n (%); PTL, platelets (× 10^9^/L), n (%); HB, hemoglobin (g/dl), n (%); NEU, neutrophils (× 10^9^/L), n (%).

APL relapse case: a 52-year-old patient diagnosed with acute promyelocytic leukemia (M3). Immunophenotypic analysis of bone marrow aspirate revealed the presence of a cluster of blasts (47%) positive for CD45^+^ dim, CD38^+^, CD71^+^ dim, CD117^+^ dim, CD13^+^, CD33^+^, CD64^+^, CD123^+^, cMPO+, CD2^−^, CD4^−^, CD11b-, CD34^−^, CD56^−^, and HLA-DR-. The patient was positive for t (15; 17) (q22; q21) PML-RAR alpha. The patient tested negative for inv (16) (p13; q22) CBFB-MYH11; t (8; 21) (q22; q22) AML1-ETO; NPM1; FLT3 ITD; CEBPA mutations.

In APL cases with no relapse history, immunophenotypic analysis of bone marrow aspirates revealed the presence of a cluster of blasts in a range of 20 to 60% in patients. All patients were positive for t (15; 17) (q22; q21) PML-RAR alpha and negative for inv (16) (p13; q22) CBFB-MYH11; t (8; 21) (q22; q22) AML1-ETO; NPM1; FLT3 ITD; CEBPA mutations (see Table 2).

### Treatment of Patients

All acute promyelocytic leukemia patients (with +/− relapse history) received induction therapy (PETHEMA/HOVON LPA 2005; intermediate risk) of oral all-trans retinoic acid, ATRA (45 mg/m^2^/day, starting from day 1 until clinical remission (CR) (PML-RAR alpha <0.001%; blasts <5% in the bone marrow; NEU >1.5 × 10^9^/L; PLT>100 × 10^9^/L, no leukemic blasts or promyelocytes in the peripheral blood, no extra medullary leukemia), but not more than 90 days of treatment) plus Idarubicin, IDA (12 mg/m^2^/day, starting from days 2, 4, 6, and 8). After achieving remission, induction therapy was followed by three monthly cycles of consolidation therapy: Cycle 1—IDA 7 mg/m^2^/d [days 1–4], ATRA 45 mg/m^2^/d [days 1–15), Cycle 2—mitoxantrone 10 mg [days 1–3], ATRA 45 mg/m^2^/d [days 1–15], and Cycle 3: IDA 12 mg/m^2^/d [days 1–2], ATRA 45 mg/m^2^/d [days 1–15]. After consolidation, maintenance therapy was applied and consisted of 6-mercaptopurine, 50 mg/m^2^ [daily]; methotrexate, 15 mg/m^2^ [weekly]; and ATRA, 45 mg/m^2^/d [for 15 days every 3 months]. Maintenance therapy was continued for 15 months (see Tables 1, 2).

Treatment of APL relapse case: when relapse was diagnosed, Trisenox (0.15 mg/kg/d) induction therapy was administered for 60 days, after which PML-RAR alpha was 0.15%. Then, consolidation therapy with Trisenox 0.15 mg/kg/d was administered for 25 days, and molecular remission was achieved. Autologous stem cell transplantation was planned to prolong remission.

### Reverse Transcription quantitative PCR

Total RNA was purified using the TRIzol reagent (Invitrogen, Carlsbad, CA, United States). cDNA was synthesized using Maxima First Strand cDNA Synthesis Kit for RT-qPCR (Thermo Fisher Scientific, Waltham, MA, United States), and qPCR was performed using Maxima SYBR Green/ROX qPCR Master Mix (2×) (Thermo Fisher Scientific) on the Rotor-Gene 6000 system (Corbett Life Science, QIAGEN, Hilden, Germany). Primers sequences (Metabion international AG, Planegg/Steinkirchen, Germany) are outlined in Supplementary Table S1, and the reaction conditions are described according to Borutinskaite et al. (2018). mRNA levels were normalized to GAPDH expression. Relative gene expression was calculated using the ∆∆Ct method (Schmittgen and Livak, 2008).

### Protein Isolation, Gel Electrophoresis, and Western Blot Analysis

Cell lysates were prepared as described previously (Vitkeviciene et al., 2019). Proteins were fractionated in 7.5–15% SDS-PAGE gradient electrophoresis gel and transferred on the PVDF membrane. Primary antibodies against ATM (mouse, clone 6F-H2) (Thermo Fisher Scientific, Waltham, MA, United States), Phospho-ATM (Ser 1981) (Abcam) (dilution ratio 1:15000), SUZ12 (Cell Signaling Technology) (dilution ratio 1:1000), H3K27me3 (rabbit, polyclonal) (Millipore, Billerica, MA, United States), H3K14Ac (rabbit, polyclonal) (Millipore, Billerica, MA, United States), H4 hyper Ac (rabbit, polyclonal) (Millipore, Billerica, MA, United States), EZH2 (Cell Signaling Technology, Danvers, MA, United States) (dilution ratio 1:1000), GAPDH (mouse, clone 6C5) (Abcam, Cambridge, United Kingdom), HRP-conjugated secondary antibodies against mouse immuno-globulins (goat, polyclonal) (Agilent Dako, Santa Clara, CA, United States), and rabbit immunoglobulins (goat, polyclonal) (Agilent Dako, Santa Clara, CA, United States) were used according to the manufacturer’s instructions. GAPDH was used as a loading control. “Clarity Western ECL Substrate” (BioRad, Hercules, CA, United States) was used for chemiluminescent detection. Signal detection was carried out on ChemiDoc™ XRS+ System (BioRad, Hercules, CA, United States). Quantitative evaluation was performed using ImageJ software.

### Sample Preparation for Mass Spectrometry Analysis

The filter aided sample preparation (FASP) (Wisniewski et al., 2009) method was used for protein digestion prior to mass spectrometry analyses. Protein lysates were processed by the FASP using 30 k centrifugal ultrafiltration units (Millipore) operated at 10 000 g. Briefly, the sample was diluted with 200 μl of 8 M urea (pH 8.5), placed in a filter unit, centrifuged, and washed two times with 100 μl of 8 M urea. Then, 100 μl of 55 mM iodoacetamide was added to the filters, and samples were incubated for 20 min. Filters were washed twice with 100 μl of 8 M urea, followed by two washes with 100 μl of 50 mM NH_4_HCO_3_ pH 8.0. Protein digestion was then performed by adding trypsin in 50 μl of 50 mM NH_4_HCO_3_ at an enzyme to protein ratio of 1:100 and incubating overnight at 37°C. Peptides were collected from the concentrators by centrifugation at 10,000 *g* for 10 min and additionally eluted using 20% CH_3_CN. The eluates were combined, acidified with 10% CF_3_COOH, and the peptides were dried in a speed vacuum for 2 h at 45°C. The lyophilized peptides were re-dissolved in 0.1% formic acid.

### LC-MS Based Protein Identification

Liquid chromatographic (LC) analysis was performed on a Waters Acquity ultra performance LC system (Waters Corporation, Wilmslow, United Kingdom). Peptide separation was performed on an ACQUITY UPLC HSS T3 250 mm analytical column. Data were acquired using Synapt G2 mass spectrometer (MS) and Masslynx 4.1 software (Waters Corporation) in positive ion mode using data-independent acquisition (UDMSE). Raw data were lock mass-corrected using the doubly charged ion of [Glu1]-fibrinopeptide B (m/z 785.8426 [M+2H]^2+^). Raw data files were processed and searched using ProteinLynx Global SERVER (PLGS) version 3.0.1 (Waters Corporation, United Kingdom). Data were analyzed using trypsin as the cleavage protease, one missed cleavage was allowed, and fixed modification was set to carbamidomethylation of cysteines, the variable modification was set to oxidation of methionine. Minimum identification criteria included 1 fragment ion per peptide, 3 fragment ions, and one peptide per protein. The following parameters were used to generate peak lists: 1) low energy threshold was set to 150 counts, 2) elevated energy threshold was set to 50 counts, 3) intensity threshold was set to 750 counts. UniprotKB/SwissProt human database was used for protein identification. Protein quantification was calculated using the ISOQuant software.

### Statistical Analysis

Data are expressed as mean ± standard deviation (S.D.). Two-tailed Student’s t-test was used to determine the significance of the difference between groups of treated and untreated samples; significance was set at *p* ≤ 0.05 (*).

## Results

In this study, we analyzed the proteomic and gene expression signatures of samples collected from patients positive for t (15; 17) (q22; q21) PML-RAR alpha who entered molecular remission after standard treatment with all-trans retinoic acid, idarubicin, and mitoxantrone or relapsed after such treatment. All patients enrolled in this study were negative for inv (16) (p13; q22) CBFB-MYH11; t (8; 21) (q22; q22) AML1-ETO; NPM1; FLT3 ITD; CEBPA (see Tables 1, 2). One patient relapsed after standard treatment and reached molecular remission only after treatment with arsenic trioxide (Trisenox) (see Table1).

### Proteomic Analysis of Leukemia Patients

We performed mass spectrometry analysis of bone marrow samples of the relapsed patient (patient No.8, Table 1) at a few time points: diagnosis stage, after standard treatment with all-trans retinoic acid, idarubicin and mitoxantrone, at the relapse stage, and after treatment with Trisenox (for more details see Table 1).

**FIGURE 1:**
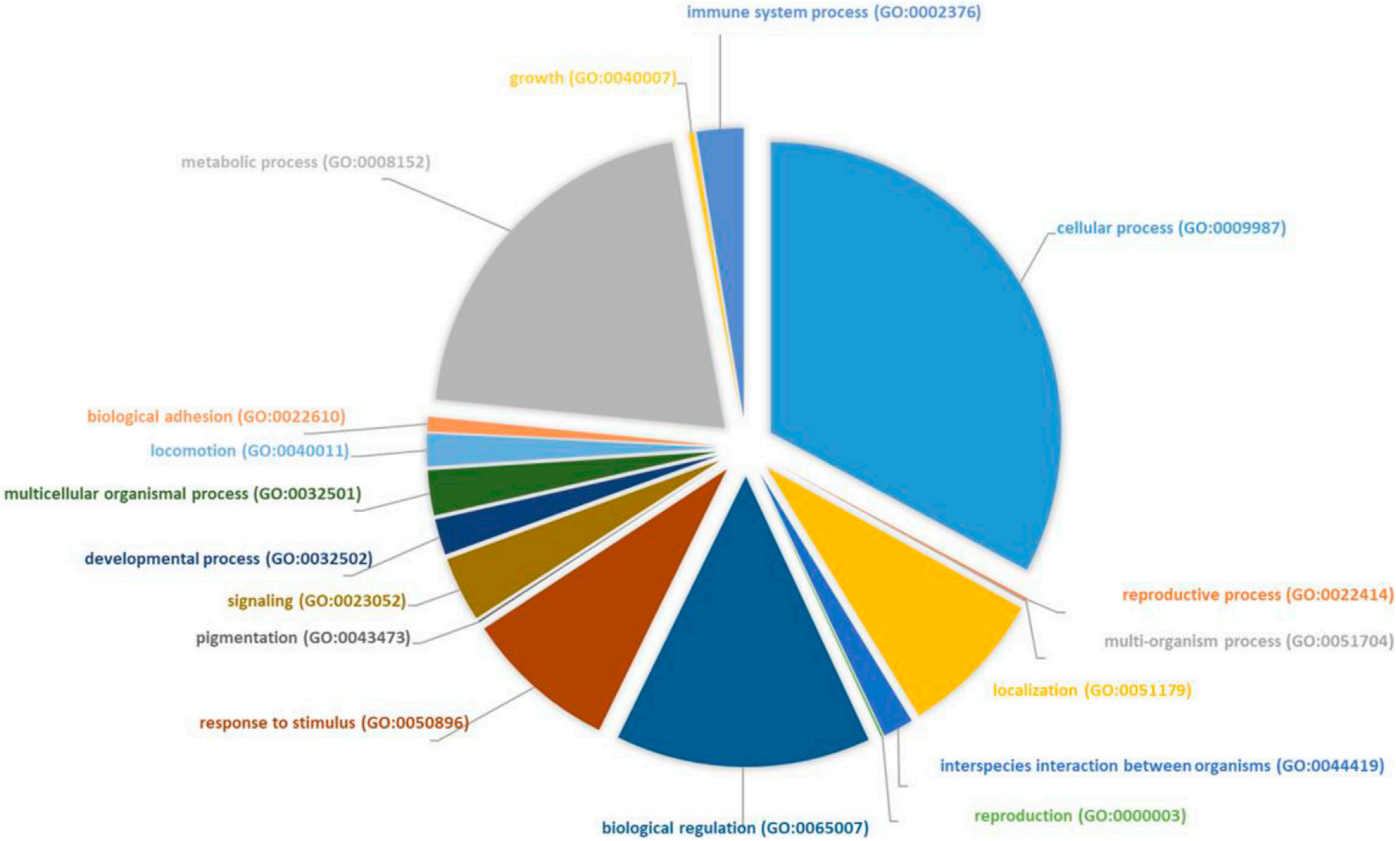
Identified proteins by mass spectrometry are separated according to their involvement in the biological process. Analysis was performed using the PANTHER analysis tool.

**FIGURE 2:**
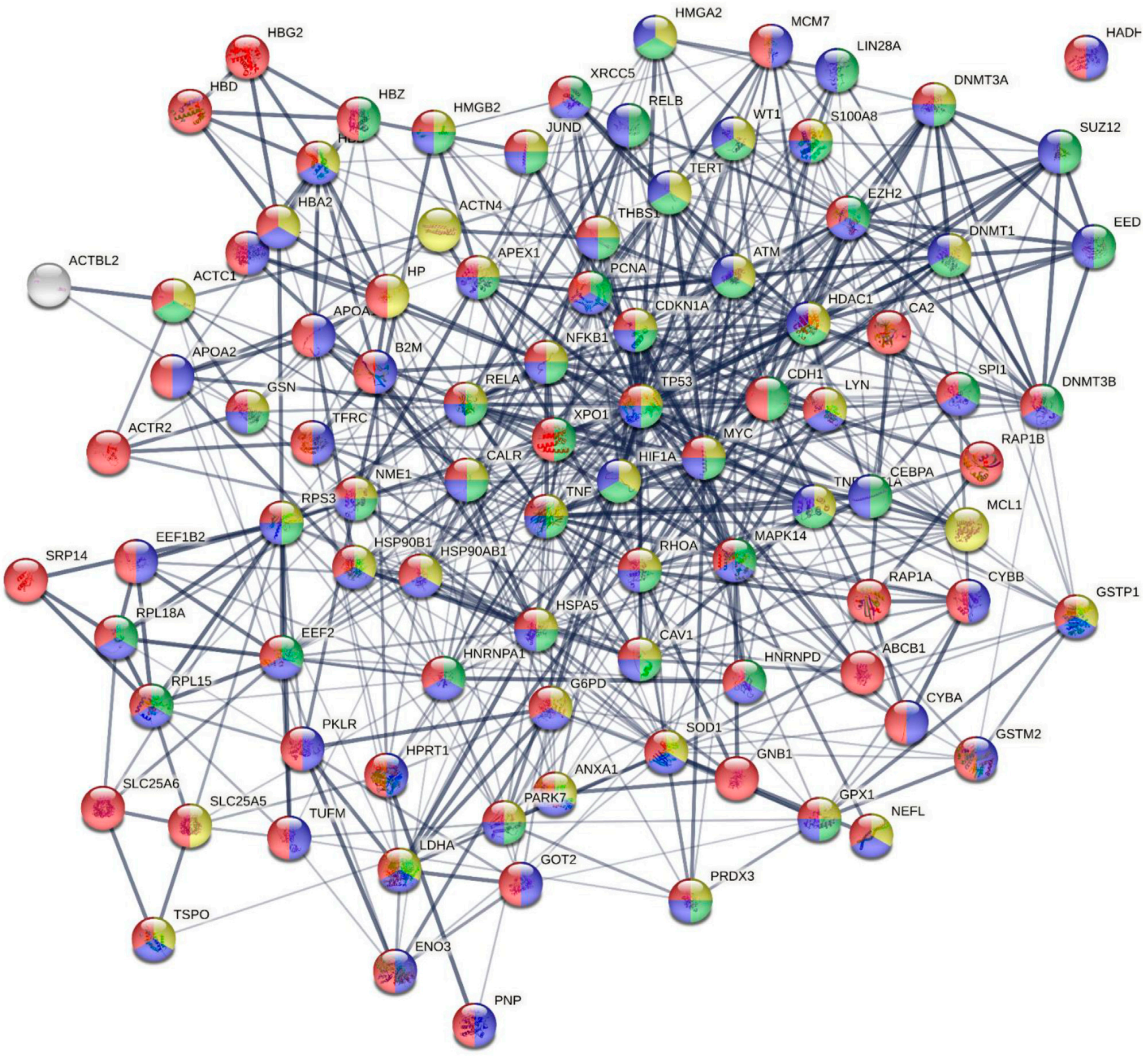
Proteins involved in drug response process interaction network. Proteins whose expression changed were displayed using the functional protein association network analysis tool STRING (https://string-db.org/). Blue, metabolic process (GO: 0008152); red, response to drug (GO: 0042493); yellow, regulation of cell death; green, regulation of gene expression (GO: 0010468). A list of the proteins is presented in Supplementary Tables S2, S3.

In total, we identified around 800 proteins, which can be divided into groups based on the biological process they are involved in (see Supplementary Table S2, Figure 1). We found that most identified proteins (450 identified proteins) are involved in the cellular process: 23.7% proteins (of 450 proteins) important for cellular metabolic process, 16.2% cellular component organization or biogenesis, 8.8% cellular response to stimulus and 9% cell cycle/cell death. Different identified enzymes can be important metabolic processes within the cell-like primary metabolic process, organic substance metabolic process, etc. (Figure 1, Supplementary Table S2). In total, 194 proteins belong to the biological regulation process. We identified 120 proteins that are important in immune system processes such as neutrophil degranulation, neutrophil-mediated immunity, myeloid leukocyte mediated immunity, leukocyte degranulation, immune response, myeloid cell activation, and immune response. Also, we identified 66 proteins that are important for drug response within the cell, like annexin A1, apolipoprotein A, carbonic anhydrase 2, gelsolin, heterogeneous nuclear ribonucleoproteins, heat shock proteins, and others (Supplementary Tables S2, S3). All these proteins were differently expressed during the treatment period. Partly these proteins are also involved in metabolic regulation, cell death, and regulation of gene expression processes (Figure 2). We detected that 38 proteins were upregulated at the molecular remission time point versus relapse (Rel/Mol.remission <1), and the other 28 were downregulated after treatment with arsenic trioxide (Rel/Mol.remission >1) (Supplementary Table S3). However, when analyzing the results of the proteomic signatures at different stages of treatment (diagnosis, after standard treatment, relapse, and molecular remission), we did not detect proteins that could reflect the regular course of the disease and predict the outcome of the disease.

### Expression of Genes Involved in Response to Drug Treatment and Regulation of Apoptosis, Cell Proliferation Processes

**FIGURE 3:**
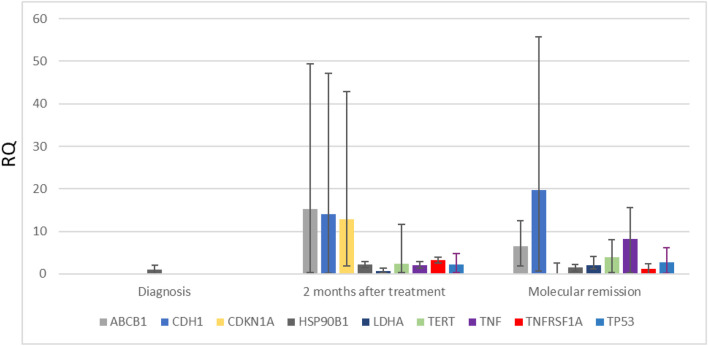
Expression of genes involved in response to drug treatment and regulation of apoptosis and cell proliferation processes (ABCB1, CDH1, CDKN1A, HSP90B1, LDHA, TERT, TNF alpha, TNFRSF1A, and TP53) in APL patients (*n* = 8). Gene expression changes were measured by the reverse transcription quantitative polymerase chain reaction (RT-qPCR) method. GAPDH was used as a “housekeeping” gene; results are presented as changes in comparison to diagnosis stage; results are mean ± SD (*n* = 3); *p ≤ .05, calculated by the Student t test. RQ, relative quantification, 2−ΔΔCt.

One of the identified proteins by mass spectrometry analysis was lactate dehydrogenase (LDHA). This protein is involved not only in the drug response process but also in metabolic regulation of cell death, cell proliferation, and gene expression processes. The LDHA protein level detected by mass spectrometry analysis gradually increased during treatment and continued to increase at the relapse and after treatment with arsenic trioxide (Supplementary Tables S2, S3). The gene expression revealed no significant changes in expression during treatment in patients with no relapse history (Figure 3) or in patients with a relapse history (Tables 1, 2). Another protein identified by mass spectrometry analysis was endoplasmin (HSP90B1). This protein is mostly involved in the regulation of apoptosis and cell proliferation. Gene expression was slightly upregulated during treatment in patients with no relapse history (Figure 3).

We also analyzed gene expression of genes that are important for drug response and cell apoptosis/proliferation processes like ABCB1, CDH1, CDKN1A, TERT, TNFalpha, TNFRSF1A, and TP53 (Figure 3). After 2 months of standard treatment, we detected upregulation of ABCB1, CDH1, CDKN1A, and TNFRSF1A in all patients. At the molecular remission stage (after 2 years), only CDH1, TERT, and TNFalpha genes were upregulated in comparison with the point at 2 months of treatment (Figure 3). Due to the high dispersion of the results between patients, no reliable changes in ABCB1, CDH1, CDKN1A, TERT, TNFalpha, TNFRSF1A, and TP53 gene expression have been found (Figure 3).

### Expression of Genes and Proteins Involved in Drug Treatment and Regulation of Gene Expression Processes

**FIGURE 4:**
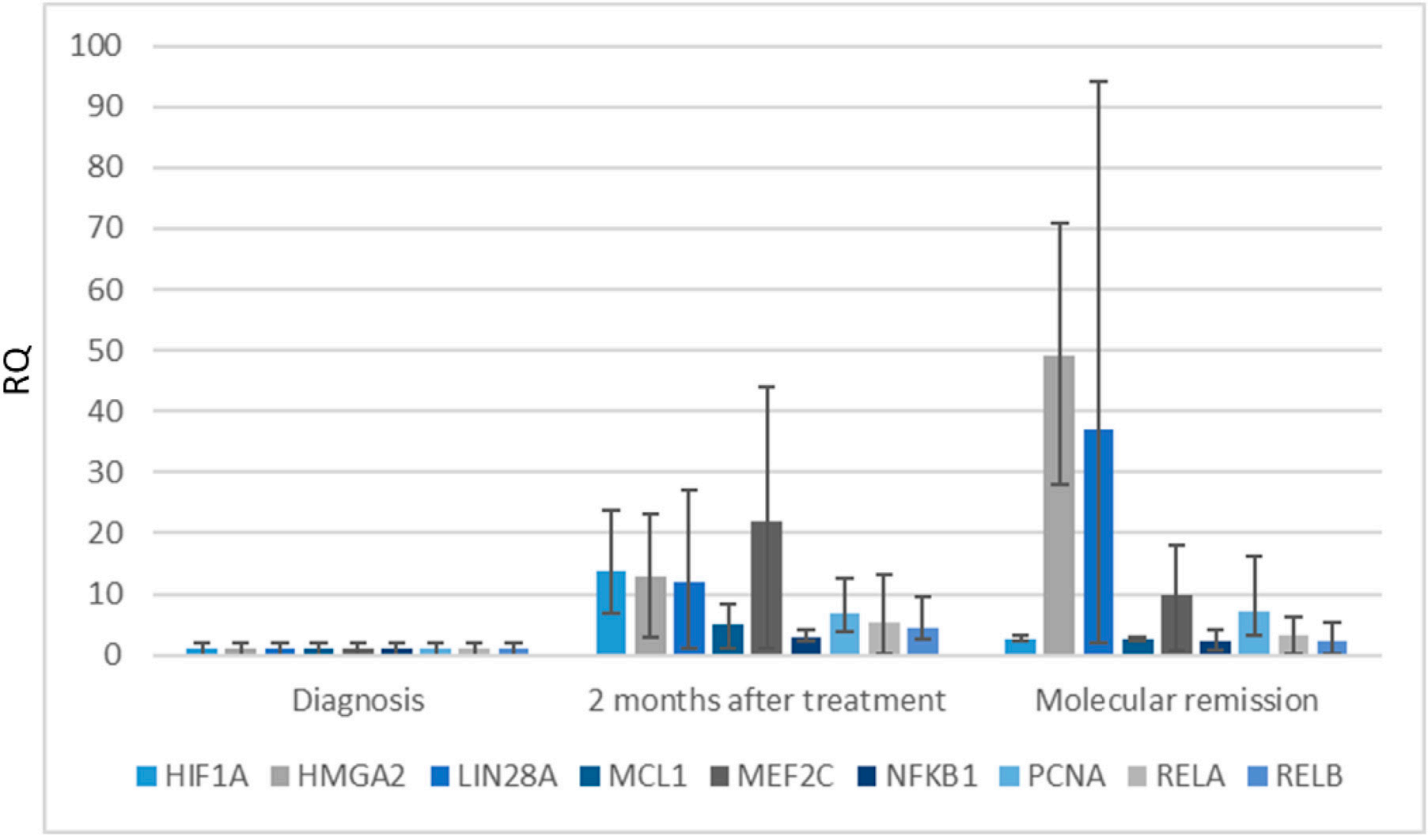
Expression of genes involved in response to drug treatment processes (HIF1A, HMGA2, LIN28A, MCL1, MEF2C, NFKB1, RELA, and RELB) in APL patients (*n* = 8). Gene expression changes were measured by the reverse transcription quantitative polymerase chain reaction (RT-qPCR) method. GAPDH was used as a “housekeeping” gene; results are presented as changes in comparison to diagnosis stage; results are mean ± SD (*n* = 3); *p ≤ .05, calculated by the Student t test. RQ, Relative quantification, 2−ΔΔCt.

One of the identified proteins by mass spectrometry analysis was proliferating cell nuclear antigen (PCNA). Gene expression analysis revealed that during treatment of APL patients, it was observed upregulation of PCNA (Figure 4). Also, we performed expression analysis of other genes like NFKB1, RELA, RELB, and others that are in the PCNA interaction network (Figure 2). We detected that HMGA2, LIN28A, and MEF2C gene expression were upregulated at the molecular remission stage in comparison with the diagnosis stage (Figure 4). However, the expression of these genes was very different from patient to patient, and we did not notice any significant correlation between molecular remission and gene expression.

**FIGURE 5:**
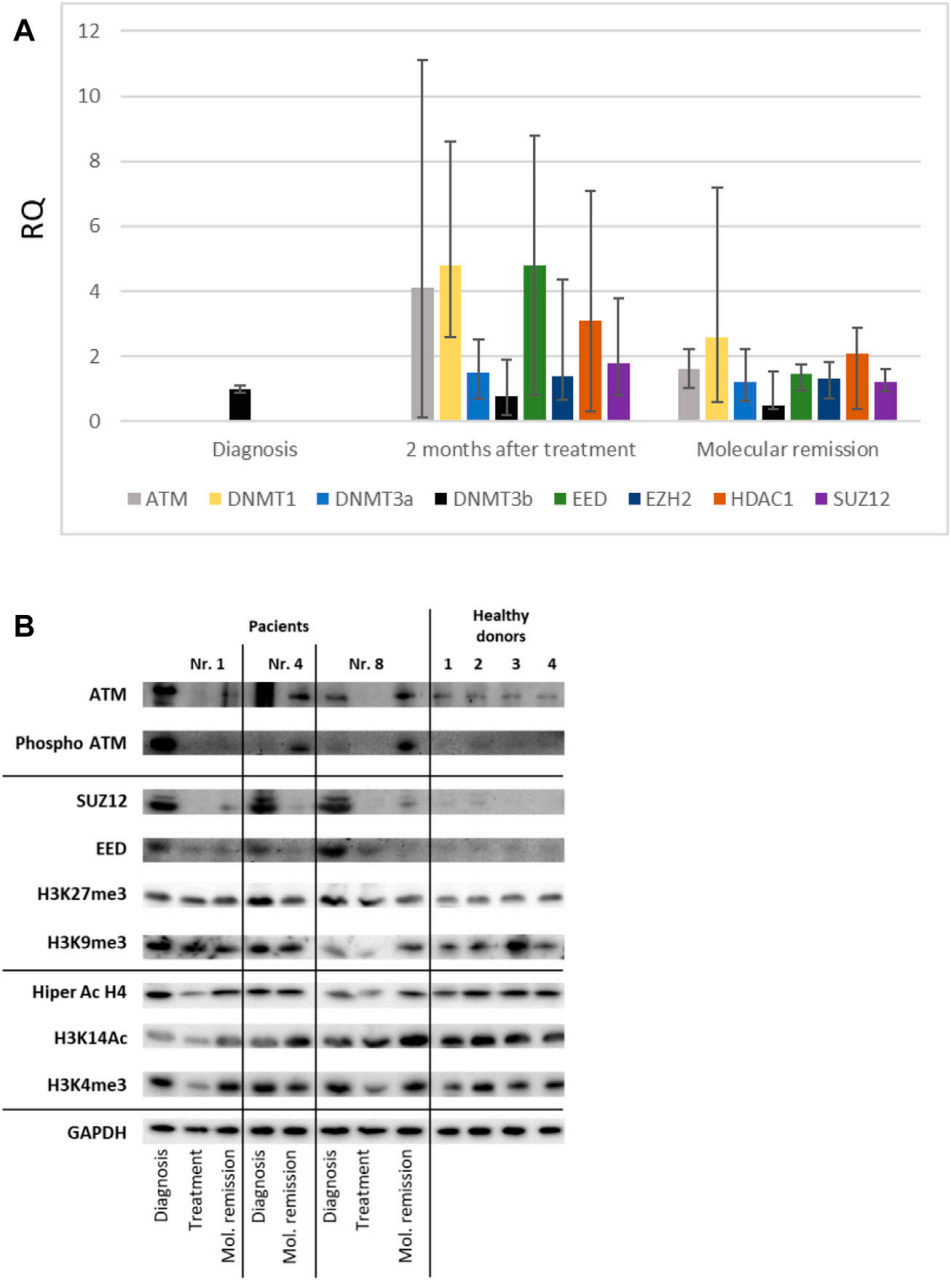
Expression of genes and proteins involved in response to drug treatment processes (ATM, DNMT1, DNMT3a, DNMT 3b, EED, EZH2, HDAC1, and SUZ12) in APL patients (*n* = 8). **(A)** Gene expression changes were measured by the reverse transcription quantitative polymerase chain reaction (RT-qPCR) method. GAPDH was used as a “housekeeping” gene; results are presented as changes in comparison to diagnosis stage; results are mean ± SD (n = 3); *p ≤ .05, calculated by the Student t-test. **(B)** ATM, phospho-ATM, SUZ12, EED, H3K27me3, H3K9me3, hyperacetylated H4, H3K14Ac, and H3K4me3 changes were assessed using immunoblot; GAPDH was used as a loading control. The experiment was repeated at least twice; representative results are shown. RQ, relative quantification, 2−ΔΔCt.

Analyzing genes involved in epigenetic regulation of transcription, we found that ATM gene expression was significantly upregulated after treatment of APL patients. However protein expression analysis revealed the downregulation of ATM. The phosphorylation of ATM was upregulated in two patients after treatment (Figures 5A,B). The proteins of the polycomb complex, EED, and SUZ12 were also downregulated after treatment of APL patients (Figure 5B) and at the molecular remission stage reached the same low expression level as in donor bone-marrow samples. However, EED and SUZ12 gene expression levels were upregulated (Figure 5A). As the PRC2 complex is responsible for the tri-methylation of H3K27, the H3K27me3 protein level was also assessed; a slight decrease in the amount of tri-methylation of H3K27 after treatment was observed compared to the amount of this modification at the time of diagnosis (Figure 5B). Western blot results showed that the total amount of H3K9me3 in bone marrow samples, both during the diagnosis of APL and in healthy donors, did not differ (Figure 5B). Also, we studied changes in histone acetylation—HiperAcH4 and H3K14Ac—in bone marrow samples. We detected no changes in hyper-acetylated H4 histone, but H3K14 acetylation increased as the patient recovered and reached a similar level to that of healthy donors (Figure 5B). Differences in the active chromatin marker H3K4me3 between bone marrow samples are not visible at diagnosis, after treatment, or in healthy donors (Figure 5B). Also, we detected DNMT1, DNMT3a, DNMT3b, and HDAC1 upregulation after treatment of APL patients (Figure 5A). But due to the high dispersion of the results from patient to patient, no reliable conclusions can be drawn.

### Expression of CALR, CAV1, CEBPA, MYC, and WT1 Genes in APL Patients

**FIGURE 6:**
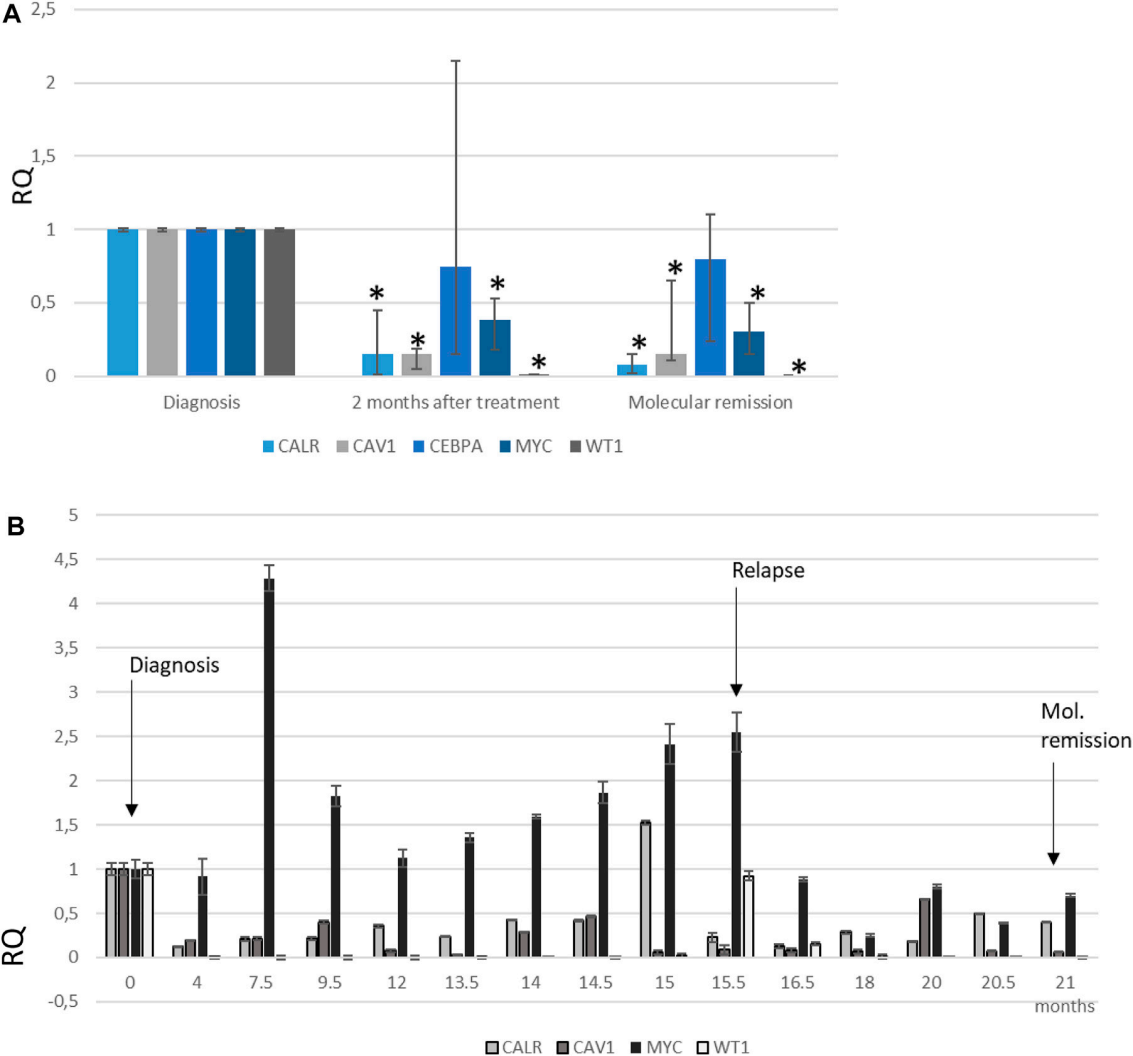
Expression of calreticulin (CALR), caveolin1 (CAV1), MYC, and WT1 genes in APL patients (*n* = 8). Gene expression changes were measured by the reverse transcription quantitative polymerase chain reaction (RT-qPCR) method. **(A)** APL patients with no relapse history **(B)** APL relapsed patient. GAPDH was used as a “housekeeping” gene; results are presented as changes in comparison to diagnosis stage; results are mean ± SD (*n* = 3); *p ≤ 05, calculated by the Student t test. RQ, relative quantification, 2−ΔΔCt.

In this study, we detected a 100-fold downregulation of WT1 gene expression after 2 months of APL patients’ treatment with no relapse history (Figure 6A). After 2 years of treatment, when complete remission occurred, we detected a 1000-fold decrease in WT1 gene expression in comparison with samples at diagnosis. We did not observe WT1 gene expression in healthy donor bone marrow samples (data not shown). In the case of an APL patient with a relapse, WT1 gene expression was dramatically downregulated to an undetectable level after 7.5 months of treatment (Figure 6B), when molecular remission was achieved (PML-RAR alpha mRNA was negative/<0.001%). After 13.5 months from diagnosis,a bone marrow aspirate revealed the presence of PML-RARαfusion transcript and WT1 gene expression started to grow. Only at 15.5 months after diagnosis, relapse was diagnosed due to blood parameters: PML-RARα fusion transcript (21.9%), 22% blasts, WBC 1.42 × 10^9^/L, NEU 0.64 × 10^9^/L, HGB 10^6^ g/L, and PLT 16 × 109/L. The patient was hospitalized and treated with Trisenox until molecular remission was achieved. WT1 gene expression started to increase 2 months before the disease relapsed (Figure 6B). Almost the same WT1 expression levels were observed at relapse (RQ = 0.92) and at the diagnosis (RQ = 1). The relapsed patient was hospitalized and treated with Trisenox for 3 months. WT1 gene expression again was downregulated and reached 0.0008 (1000-fold change) after 5 months of treatment with Trisenox (Figure 6B).

One of the identified proteins by mass spectrometry analysis was Calreticulin (CALR). We found significant downregulation after treatment and at the molecular remission stage in all APL patients (Figure 6A). In the case of APL patient with a relapse, CALR gene expression was downregulated during the first 4 months of treatment and then started to increase. At the 15-month point, CALR gene expression was upregulated, but after 15.5 month CALR gene expression started to decrease (Figure 6B). Another significantly downregulated gene – Caveolin (CAV1) was observed after treatment of APL patients (Figure 6A). Also, in the APL relapse case, we revealed downregulation of the CAV1 gene (Figure 6B). We studied CEBPA gene expression and found downregulation in APL patients; however, the large dispersion of the results in the patient group does not lead to reliable conclusions (Figure 6A).

In our study, MYC gene expression analysis in APL patients with no relapse history revealed that MYC was overexpressed at disease diagnosis and then significantly decreased up to 3-fold after treatment (Figure 6A). During analysis of the relapse case, it was detected that MYC expression did not decrease after treatment, but the expression was upregulated just before the relapse point. After treatment with Trisenox, MYC expression was downregulated (Figure 6B).

## Discussion

Since combined treatment of anthracyclines like Idarubicin or Daunorubicin with all-trans retinoic acid for the treatment of APL was introduced, around 85% of patients can achieve complete remission (Tomita et al., 2013; Westermann and Bullinger, 2021). However, patients resistant to treatment, i.e., those with resistance to ATRA and/or anthracyclines, are recognized as a clinically significant problem. A majority of these patients relapse within a three-year period, and long-term survival still remains poor. Comprehensive proteomic and genomic analysis using patient samples can help predict disease, select effective targeted drugs, and develop novel APL treatment strategies.

To identify novel possible markers that might be important for the prediction of APL outcome and relapse possibility, we performed the patient’s gene and protein expression analysis at diagnosis, during treatment, and at complete remission. All these patients possessed PML-RAR alpha and were treated with cytotoxic drugs, Idarubicin, and ATRA. Patients have reached complete remission and have had no detectable relapse. Also, we used mass spectrometry analysis to identify proteins that may be important for the prediction of relapse in APL patients. For that purpose, we analyzed bone marrow samples of APL patients with a relapse history.

Analyzing APL relapse patient samples at different stages of disease like diagnosis, relapse, and molecular remission, we detected around 800 proteins involved in different cellular processes like cellular metabolic processes (nucleolin, neutrophil cytosol factor 2, tyrosine-protein kinase HCK, etc.), cellular component organization or biogenesis (few types of histone, different types of immunoglobulin, heat shock proteins, and ribosomal proteins, etc.), cellular response to stimulus (peroxiredoxin-4, different types of immunoglobulin, heat shock proteins, and neutrophil defensins, etc.) and cell cycle/cell death (tubulin, septins, nucleophosmin, subunits of the proteasome activator complex, cytochrome c, annexins, heat shock proteins, hypoxia upregulated protein 1, etc.). Different enzymes like glycogen phosphorylase, ornithine aminotransferase, fumarate hydratase, triosephosphate isomerase, hexokinase-1 can be important for primary metabolic processes, organic substance metabolic processes, etc. Other proteins like different types of heterogeneous nuclear ribonucleoproteins, heat shock proteins, and immunoglobulins, kinases, and others were identified and belong to the biological regulation process. We identified proteins like copine, annexins, heat shock proteins, bone marrow glycoprotein that are important in immune system processes like neutrophil degranulation, neutrophil-mediate immunity, myeloid leukocyte mediated immunity, leukocyte degranulation, immune response, myeloid cell activation, and immune response. It has been shown in the literature that comprehensive bioinformatics analysis can be used to build an applicable model to predict the survival probability of AML patients in clinical use. The authors showed that FLT3, CD177, and TTPAL genes were involved in the prognosis of AML. They also revealed that calreticulin (CALR) gene expression was upregulated in AML patient samples (Qu et al., 2020). Another study of the proteomic profile in APL vs. AML cases revealed that most proteins that were higher expressed in APL were involved in the pro-apoptotic pathway, linked to higher proliferation, which may suggest a more pro-apoptotic tendency of those cells (Hoff et al., 2019).

We identified proteins that are important for drug response within the cell-like annexin A1, apolipoprotein A, carbonic anhydrase 2, gelsolin, heterogeneous nuclear ribonucleoproteins, heat shock proteins, and others. These proteins are also involved in metabolic, gene expression regulation, and apoptotic processes. It is known that Idarubicin inserts itself into DNA and blocks enzyme topoisomerase II activity, leading to damage of DNA and cell membrane, G2/M cell-cycle block and p53-mediated apoptosis (Watanabe et al., 1997). ATRA causes terminal differentiation of leukemic promyelocytes both *in vitro* and *in vivo* studies. It was shown that ATRA induces complete remission in the majority of patients with APL (Asou et al., 1998; Morgan et al., 2012). However, drug resistance in APL is still not clear and can be the result of multiple factors like drug resistance-related protein (ABCB1) and enzymes (glutathione-S-transferase (GST), topoisomerase II, protein kinase C), genetic alterations like Fms-like tyrosine kinase 3 (FLT3), Wilms tumor (WT1), IDH1, TP53, ASXL1, DNMT3A, CEBPA, IDH2, PTPN11, and miRNA alterations or drug resistance-related signal pathways like PI3K/AKT, NF-κB, PLCγ/Raf/Erk, and PKCα (Mahadevan and List, 2004; Zhang et al., 2019).

In this study, genes such as ABCB1, ATM, CALR, CAV1, CDH1, CDKN1A, CEBPA, DNMT1, DNMT3a-3b, EED, EZH2, HDAC1, HIF1A, HMGA2, HSP90B1, LDHA, LIN28A, MCL1, MEF2C, MYC, NFKB1, RELA, RELB, PCNA, SUZ12, TERT, TNF, TNFRSF1A, TP53, and WT1 that are involved not only in drug response process but also in cell cycle, apoptosis proliferation, metabolic, and epigenetic regulation processes were investigated. We observed after 2 months of standard treatment with all-trans retinoic acid and idarubicin, upregulation of ABCB1, CDH1, CDKN1A, TNFRSF1A, and epigenetic regulators like ATM, DNMT1, HDAC1, polycomb complex components EED, and SUZ12 in all APL patients. Due to the high dispersion of the results between patients, we can notice that monitoring the expression changes of these genes during the course of treatment of APL is not useful for prognostic purposes.

We observed that only WT1 (Wilms tumor protein), CALR (calreticulin), CAV1 (caveolin1), and proto-oncogene Myc (MYC) gene expression in all APL patients with no relapse history was significantly downregulated after treatment with all-trans retinoic acid and idarubicin.

In the case of an APL patient with a relapse WT1 gene expression was dramatically down regulated to an undetectable level after treatment, but after 13.5 months from diagnosis, WT1 gene expression started to grow. This upregulation correlated with presence of PML-RARalpha fusion transcript in bone marrow aspirate. We detected that WT1 gene expression started to increase 2 months before the disease relapse and almost reached WT1 expression levels at the diagnosis point. WT1 gene expression was down regulated and reached 0.0008 (1000-fold change) after treatment with Trisenox. Our findings correlate with data in the literature when it was shown that WT1 is overexpressed in mRNR and protein levels in solid cancers, such as brain, breast, cervical, colon, glioblastoma, and others, and blood cancers, such as acute and chronic leukemia, myeloma, and myelodysplastic syndrome (Silberstein et al., 1997). Also, our findings support previous data that WT1 expression levels have prognostic value in patients with APL on overall survival of responders to induction therapy and the determination of expression levels of WT1 might contribute to risk stratification in the future (Hecht et al., 2015). In breast cancer, WT1 controls the expression of genes encoding components of the insulin-like growth factor and transforming growth factor β signaling systems that are important for growth and differentiation of the mammary gland (Nebbioso et al., 2017). The WT1 transcript was detected in 80–86% of ALL patients and in 93% of AML patients. In contrast to acute leukemia, mononuclear cells from bone marrow or peripheral blood of healthy volunteers did not express the WT1 gene at detectable levels (Menssen et al., 1995; Padmakumar et al., 2021). Mutations in exons 7 and 9 of WT1 have been identified in acute myeloid leukemia and are related to poorer prognosis and resistance to treatment (Aref et al., 2014).

We detected overexpression of MYC at diagnosis in APL patients with no relapse history. MYC gene expression significantly decreased up to 3-fold after treatment. During analysis of a relapse case, it was detected that MYC expression did not decrease after treatment with Idarubicin and ATRA. After treatment with Trisenox, MYC expression was downregulated significantly. Recently, it was shown that c-Myc protein undergoes acetylation and c-Myc downregulation occurs *ex vivo* in primary AML samples treated with HDAC inhibitors (Lin et al., 2007). This event can lead to TRAIL activation and apoptosis. Also, it was demonstrated that c-Myc is overexpressed in drug-resistant cells at higher levels than in non-resistant cells (Knapp et al., 2003; Pan et al., 2014). Lin and co-authors have shown that c-Myc inhibitors like 10058-F4 can inhibit cell proliferation and lead to apoptosis and cell differentiation (Lin et al., 2007).

In this study, we showed that CALR gene expression levels were downregulated after treatment in all APL patient samples. The consequence of increased CALR expression in acute myeloid leukemia (AML) has been reported in a few studies (Schardt et al., 2010; Parka et al., 2015). It was reported that there was a weak positive correlation between CALR mRNA level and bone marrow blast percentage (Parka et al., 2015). Caveolin (CAV1) is another significantly downregulated gene after treatment of APL patients with idarubicin and ATRA. It was showed by other authors that caveolin-1 and MDR-1 (ABCB1) can interact physically, or can be involved in the same aberrant pathway(s) activated during MDR-1 upregulation (Pang et al., 2004; Wang et al., 2021). It was demonstrated that there was a relationship between the overexpression of CAV1 and poor prognosis in CLL (Gilling et al., 2012). Some previous reports have demonstrated that CAV1 acts as both a tumor suppressor and an oncogene (Trimmer et al., 2010; Korakiti et al., 2020). The studies also revealed that caveolin-1 and c-Myc are favorable molecular targets in tumor cells and metastasis (Luanpitpong et al., 2020). In our studies, we demonstrated that these two genes’ expressions are very important for APL patients’ treatment prognosis.

## Conclusion

Calreticulin, caveolin1, MYC, and WT1 can be potential markers associated with the pathology, thereby revealing the potential value of this approach for a better characterization of the prediction of APL outcomes.

## Data Availability

The data presented in this study are available on request from the corresponding author.
