# Supplementary material for: Genetic and Epigenetic Signatures in Acute Promyelocytic Leukemia Treatment and Molecular Remission
Source: Front Genet. 2022 Apr 12;13:821676. doi: 10.3389/fgene.2022.821676 (PMC9039054; doi:10.3389/fgene.2022.821676)
Supplement: Supplementary file 2 [file Table1.docx]

**Supplementary Table 1.** Primers used for RT-qPCR analysis.

| **Gene** | **Uniprot number** | **Protein name** | **Primers** |
| --- | --- | --- | --- |
| ABCB1 | P08183 | ATP-dependent translocase ABCB1 | F-GTCTGGACAAGCACTGAAA  R-AACAACGGTTCGGAAGTTT |
| ATM | Q13315 | Serine-protein kinase ATM | F- CTCTGAGTGGCAGCTGGAAGA  R- TTTAGGCTGGGATTGTTCGCT |
| CALR | P27797 | Calreticulin | F-AGTTCCGGCAAGTTCTACGG  R- ACAGAGCATAAAAGCGTGCAT |
| CAV1 | Q03135 | Caveolin-1 | F-CACATCTGGGCAGTTGTACC  R-CACAGACGGTGTGGACGTAG |
| CDH1 | P12830 | Cadherin-1 | F- TCCCATCAGCTGCCCAGAAAA  R- TGACTCCTGTGTTCCTGTTA |
| CDKN1A | P38936 | Cyclin-dependent kinase inhibitor 1 | F- GGCAGACCAGCATGACAGATT  R- GCGGATTAGGGCTTCCTCT |
| CEBPA | P49715 | CCAAT/enhancer-binding protein alpha | F- GCTCGCCATGCCGGGAGAACT  R- TGCAGGTGGCTGCTCATCGG |
| DNMT1 | P26358 | DNA (cytosine-5)-methyltransferase 1 | F- CCTGCTTCAGCGTGTACTGT  R- ATCGGCTTTGCTGCTGAACCAGA |
| DNMT3A | Q9Y6K1 | DNA (cytosine-5)-methyltransferase 3A | F- ACCCTCCAAAGGTTTACCCACCTG  R- CATACCGGGAGGGTTACCCCAGAA |
| DNMT3B | Q9UBC3 | DNA (cytosine-5)-methyltransferase 3B | F- GACTGGAACGTGCGCCTGCAGGCC  R- GAAGCGACGTACTTTCCTACCTTT |
| EED | O75530 | Polycomb protein EED | F- GTGACGAGAACAGCAATCCAG  R- TATCAGGGCGTTCAGTGTTG |
| EZH2 | Q15910 | Histone-lysine N-methyltransferase EZH2 | F- GTGGAGAGATTATTTCTCAAGATG  R- CCGACATACTTCAGGGCATCAGCC |
| HDAC1 | Q13547 | Histone deacetylase 1 | F- CAAGCTCCACATCAGTCCTTCC  R- TGCGGCAGCATTCTAAGGTT |
| HIF1A | Q16665 | Hypoxia-inducible factor 1-alpha | F -CCAACAGTAACCAACCTCAG  R -TCCTGTGGTGACTTGTCCTT |
| HMGA2 | P52926 | High mobility group protein HMGI-C | F-CCCAAAGGCAGCAAAAACAA;  R-GCCTCTTGGCCGTTTTTCTC |
| HSP90B1 | P14625 | Endoplasmin | F- GCTGACGATGAAGTTGATGTGG  R- CATCCGTCCTTGATCCTTCTCTA |
| LDHA | P00338 | L-lactate dehydrogenase A chain | F -ATGGCAACTCTAAAGGATCAGC  R -CCAACCCCAACAACTGTAATCT |
| LIN28A | Q9H9Z2 | Protein lin-28 homolog A | F -TTGTCTTCTACCCTGCCCTCT  R -GAACAAGGGATGGAGGGTTTT |
| MCL1 | Q07820 | Induced myeloid leukemia cell differentiation protein Mcl-1 | F-GTGCCTTTGTGGCTAAACACT  R-AGTCCCGTTTTGTCCTTACGA |
| MEF2C | Q06413 | Myocyte-specific enhancer factor 2C | F-CGACATGCCAGTCTCCATCC-3‘  F-AGCAGACCTGGTGAGTTTCG-3‘ |
| MYC | P01106 | Myc proto-oncogene protein | F-ATGAAAAGGCCCCCAAGGTAGTTATC  R- GTCGTTTCCGCAACAAGTCCTCTTC |
| NFKB1 | P19838 | Nuclear factor NF-kappa-B p105 subunit | F -GAAGCACGAATGACAGAGGC  R -GCTTGGCGGATTAGCTCTTTT |
| PCNA | P12004 | Proliferating cell nuclear antigen | F- GCGTGAACCTCACCAGTATGT  R- TCTTCGGCCCTTAGTGTAATGAT |
| RELA | Q04206 | Transcription factor p65 | F -ATGTGGAGATCATTGAGCAGC  R -CCTGGTCCTGTGTAGCCATT |
| RELB | Q01201 | Transcription factor RelB | F -CCATTGAGCGGAAGATTCAACT  R -CTGCTGGTCCCGATATGAGG |
| SUZ12 | Q15022 | Polycomb protein SUZ12 | F- AGGCTGACCACGAGCTTTTC  R- GGTGCTATGAGATTCCGAGTTC |
| TERT | O14746 | Telomerase reverse transcriptase | F- CGTACAGGTTTCACGCATGTG  R- ATGACGCGCAGGAAAAATG |
| TNF | P01375 | Tumor necrosis factor | F -GCTGCACTTTGGAGTGATCG  R -TCACTCGGGGTTCGAGAAGA |
| TNFRSF1A | P19438 | Tumor necrosis factor receptor superfamily member 1A | F -TCACCGCTTCAGAAAACCACC  R -GGTCCACTGTGCAAGAAGAGA |
| TP53 | P04637 | Cellular tumor antigen p53 | F- TAACAGTTCCTGCATGGGCGGC  R- AGGACAGGCACAAACACGCACC |
| WT1 | P19544 | Wilms tumor protein | F- GGCATCTGAGACCAGTGAGAA  R- GAGAGTCAGACTTGAAAGCAG |
