# Supplementary material for: Genetic and Epigenetic Signatures in Acute Promyelocytic Leukemia Treatment and Molecular Remission
Source: Front Genet. 2022 Apr 12;13:821676. doi: 10.3389/fgene.2022.821676 (PMC9039054; doi:10.3389/fgene.2022.821676)
Supplement: Supplementary file 3 [file Table3.docx]

**Supplementary Table 3.** Identified proteins by mass spectrometry analysis, proteins involved in response to drug biological process.

| **Protein description** | **Entry** | **Relapse** | **Molecular**  **remission** | **Relapse/Mol.remission** |
| --- | --- | --- | --- | --- |
|  |  |  |  |  |
| 40S ribosomal protein S3 | RS3_HUMAN | 25170 | 18756 | 1.34 |
| 60S ribosomal protein L15 | RL15_HUMAN | 15361 | 16004 | 0.959 |
| 60S ribosomal protein L18a | RL18A_HUMAN | 11365 | 11263 | 1.008 |
| Actin, alpha cardiac muscle 1 | ACTC_HUMAN | 192077 | 125658 | 1.528 |
| Actin-related protein 2 | ARP2_HUMAN | 12996 | 36207 | 0.358 |
| ADP/ATP translocase 2 | ADT2_HUMAN | 17224 | 22597 | 0.762 |
| ADP/ATP translocase 3 | ADT3_HUMAN | 5668 |  |  |
| Annexin A1 | ANXA1_HUMAN | 106737 | 174955 | 0.610 |
| Apolipoprotein A-I | APOA1_HUMAN | 18158 | 20038 | 0.906 |
| Apolipoprotein A-II | APOA2_HUMAN |  |  |  |
| Aspartate aminotransferase, mitochondrial | AATM_HUMAN | 4150 |  |  |
| Beta-2-microglobulin | B2MG_HUMAN | 46013 | 11717 | 3.927 |
| Beta-actin-like protein 2 | ACTBL_HUMAN | 1221 | 824 | 1.482 |
| Beta-enolase | ENOB_HUMAN | 58984 |  |  |
| Calreticulin | CALR_HUMAN | 258832 | 161542 | 1.602 |
| Carbonic anhydrase 2 | CAH2_HUMAN | 8995 | 22238 | 0.404 |
| Cytochrome b-245 heavy chain | CY24B_HUMAN | 10474 | 31160 | 0.336 |
| Cytochrome b-245 light chain | CY24A_HUMAN | 9863 | 11641 | 0.8472 |
| DNA replication licensing factor MCM7 | MCM7_HUMAN | 2839 | 4643 | 0.6114 |
| DNA-(apurinic or apyrimidinic site) endonuclease | APEX1_HUMAN | 14423 | 24180 | 0.5964 |
| Elongation factor 1-beta | EF1B_HUMAN | 7852 | 8526 | 0.9208 |
| Elongation factor 2 | EF2_HUMAN | 34089 | 21754 | 1.5670 |
| Elongation factor Tu, mitochondrial | EFTU_HUMAN | 57217 | 51105 | 1.1195 |
| Endoplasmic reticulum chaperone BiP | BIP_HUMAN | 90568 | 63170 | 1.4337 |
| Endoplasmin | ENPL_HUMAN | 127244 | 76278 | 1.6681 |
| Exportin-1 | XPO1_HUMAN | 5870 | 9502 | 0.6177 |
| Gelsolin | GELS_HUMAN | 39178 | 93850 | 0.4174 |
| Glucose-6-phosphate 1-dehydrogenase | G6PD_HUMAN | 8110 | 25071 | 0.3234 |
| Glutathione peroxidase 1 | GPX1_HUMAN | 8312 | 5066 | 1.6409 |
| Glutathione S-transferase Mu 2 | GSTM2_HUMAN | 3147 | 3564 | 0.8829 |
| Glutathione S-transferase P | GSTP1_HUMAN | 48366 | 93332 | 0.5182 |
| Guanine nucleotide-binding protein G(I)/G(S)/G(T) subunit beta-1 | GBB1_HUMAN | 7965 | 11637 | 0.6844 |
| Haptoglobin | HPT_HUMAN | 12021 | 24366 | 0.4933 |
| Heat shock protein HSP 90-beta | HS90B_HUMAN | 33153 | 67170 | 0.4935 |
| Hemoglobin subunit alpha | HBA_HUMAN | 362738 | 234002 | 1.5501 |
| Hemoglobin subunit beta | HBB_HUMAN | 279235 | 247224 | 1.1294 |
| Hemoglobin subunit delta | HBD_HUMAN | 57777 | 64412 | 0.8969 |
| Hemoglobin subunit gamma-2 | HBG2_HUMAN | 26218 | 53052 | 0.4941 |
| Hemoglobin subunit zeta | HBAZ_HUMAN | 176774 | 157736 | 1.1206 |
| Heterogeneous nuclear ribonucleoprotein A1 | ROA1_HUMAN | 44002 | 28935 | 1.5207 |
| Heterogeneous nuclear ribonucleoprotein D0 | HNRPD_HUMAN | 28805 | 39118 | 0.7363 |
| High mobility group protein B2 | HMGB2_HUMAN | 41864 | 49804 | 0.8405 |
| Hypoxanthine-guanine phosphoribosyltransferase | HPRT_HUMAN | 4252 | 8748 | 0.4860 |
| L-lactate dehydrogenase A chain | LDHA_HUMAN | 51254 | 68978 | 0.7430 |
| Mitogen-activated protein kinase 14 | MK14_HUMAN |  | 5002 |  |
| Neurofilament light polypeptide | NFL_HUMAN | 18154 | 7297 | 2.4879 |
| Nucleoside diphosphate kinase A | NDKA_HUMAN | 15015 | 8032 | 1.8694 |
| Parkinson disease protein 7 | PARK7_HUMAN | 33085 | 28319 | 1.1683 |
| Pyruvate kinase PKLR | KPYR_HUMAN | 9493 | 6012 | 1.5789 |
| Proliferating cell nuclear antigen | PCNA_HUMAN | 5210 | 8867 | 0.5875 |
| Protein S100-A8 | S10A8_HUMAN | 190772 | 231105 | 0.8254 |
| Purine nucleoside phosphorylase | PNPH_HUMAN | 21149 | 29185 | 0.7246 |
| Ras-related protein Rap-1A | RAP1A_HUMAN | 4645 | 9338 | 0.4974 |
| Ras-related protein Rap-1b | RAP1B_HUMAN | 20902 | 13972 | 1.495 |
| Signal recognition particle 14 kDa protein | SRP14_HUMAN | 27901 | 2147 | 12.995 |
| Superoxide dismutase [Cu-Zn] | SODC_HUMAN | 26617 |  |  |
| Thioredoxin-dependent peroxide reductase | PRDX3_HUMAN | 23079 | 17996 | 1.282 |
| Thrombospondin-1 | TSP1_HUMAN |  | 6599 |  |
| Tyrosine-protein kinase Lyn | LYN_HUMAN | 965 | 3190 | 0.302 |
| Transcobalamin-1 | TCO1_HUMAN | 1354 | 9601 | 0.141 |
| Transferrin receptor protein 1 | TFR1_HUMAN | 7318 | 8340 | 0.877 |
| Transforming protein RhoA | RHOA_HUMAN | 19201 | 31522 | 0.6091 |
| Translocator protein | TSPO_HUMAN | 8094 | 17041 | 0.4750 |
| Trifunctional enzyme subunit alpha | ECHA_HUMAN | 18791 | 11860 | 1.5844 |
| X-ray repair cross-complementing protein 5 | XRCC5_HUMAN | 17034 | 27206 | 0.6261 |
